# Creating a treatment plan report should be mandated as a minimum standard practice for patient care and QA documentation

**DOI:** 10.1002/acm2.13072

**Published:** 2020-10-26

**Authors:** Ping Xia, Arthur Olch, Yi Rong

**Affiliations:** ^1^ Department of Radiation Oncology, Taussig Cancer Center The Cleveland Clinic Foundation Cleveland OH USA; ^2^ Radiation Oncology Program at Children’s Hospital Los Angeles and Department of Radiation Oncology University of Southern California Keck School of Medicine Los Angeles CA USA; ^3^ Department of Radiation Oncology Mayo Clinic Arizona Phoenix AZ USA

## 1 INTRODUCTION

In the old days when the electric charting or three‐dimensional (3D) computed tomography (CT) simulation was not yet available, treatment plans were created on two‐dimensional (2D) x‐ray images by hand and with manual calculations. With no doubt, a paper report was considered necessary for documenting the treatment plan for patients. Fast forward to today when almost every step in patient care becomes paperless and digitally recorded, is creating a plan report still needed for patient's quality care or is it just an old habit that dies hard? Herein, we have two world‐known medical physics experts debating the topic: *Creating a treatment plan report should be mandated as a minimum standard practice for patient care and QA documentation*. Dr. Ping Xia is arguing for the proposition while Dr. Arthur Olch is arguing against.

Dr. Ping Xia is the Head of Medical Physics in the Department of Radiation Oncology at Cleveland Clinic. She is a Professor of Molecular Medicine, Cleveland Clinic Lerner College of Medicine of Case Western Reserve University. After her 14 yr tenure at University of California at San Francisco as an Assistant Professor and Associate Professor in Radiation Oncology, Dr. Xia was recruited to the Cleveland Clinic’s Department of Radiation Oncology in 2009 as the Chief Medical Physicist of Radiation Oncology at Cleveland Clinic Main Campus and regional Radiation Oncology facilities. Dr. Xia has published more than 130 peer‐reviewed articles. Along with her other colleagues at Cleveland Clinic, she recently published a treatment planning book titled as “Strategies for radiation therapy treatment planning.” She received research grants from the Department of Defense and National Cancer Institution. Dr. Xia is a Fellow of The American Association of Physicists in Medicine (AAPM).

Arthur J. Olch is Professor of Radiation Oncology and Clinical Pediatrics at the Keck School of Medicine at the University of Southern California in Los Angeles, California. He is also Chief of Physics for the Children's Hospital of Los Angeles Radiation Oncology Program. Dr. Olch received his PhD in Medical Physics from the University of California, Los Angeles. He is a Fellow of the American Association of Physicists in Medicine (AAPM). Dr. Olch has served as the Chair of the Physics Subcommittee of the Children's Oncology Group and of the AAPM Subcommittee on Quality Assurance and Outcome Improvement as well as serving as chair or member of various AAPM Task Groups. He has been a radiation therapy physicist for over 30 years and has authored or coauthored over 70 journal articles and book chapters. He is the author of the book, “Pediatric Radiotherapy Planning and Treatment.”

## 2 OPENING STATEMENTS

### 2.A Dr. Ping Xia

Upon completion of a treatment plan, creating a plan report has been standard practice for radiotherapy since the era of computerized planning began. With the integration of radiation oncology specific electronic medical record systems (RO‐EMR) and treatment planning systems (TPS), there has been some thought in our community that creating a plan report may no longer be necessary because everyone in a radiation oncology department can directly access the TPS for plan information, with the assumption that everyone is familiar with and understands the workflow of the TPS. This is a rather big assumption.

Quality assurance of a treatment plan is paramount. In a recent publication, 33% of events reported to the radiation oncology incident learning system (RO‐ILS) occurred during the processes of treatment planning and pretreatment review/verification.^1^ Treatment planning is a complex process. Any errors in this process may lead to catastrophic errors, or nondeliverable plans, causing treatment delay. Mitigation of these hazards is best accomplished by using multiple layers of plan checks/reviews,^2^ involving medical physicists, medical dosimetrists, radiation oncologists, and radiation therapists. Among these team members, many are not well‐trained to use or routinely use the TPS. Even among well‐trained users such as dosimetrists, a comprehensive and standardized treatment plan report is an excellent tool to systematically evaluate plan integrity. It is an added effort but a worthwhile effort. Additionally, all major planning systems have tools to create detailed plan reports automatically, making the effort minimal. Creating a plan report alone is a process of quality assurance, assembling critical information of the plan and forcing the planner to focus on the whole planning process.

A draft of the AAPM task group (TG) 262 report, which is charged with providing guidelines on the use of the RO‐EMR as an information repository, states that the purpose of a treatment plan report is for internal review and patient care continuity in outside departments or institutions. Accessibility to treatment plan information should be quick and easy, especially for patients who need emergent treatments. A plan report meets this requirement. When saved in a format such as PDF, a plan report also provides a durable and difficult to modify record of the plan, independent of the planning system. In the event that planning systems become inaccessible due to upgrades or software changes, a PDF plan report will still be accessible. For patient care continuity in outside institutions, it has been suggested that just‐in‐time creation of plan reports or providing DICOM data upon request could be viable options. However, data availability upon request may not always be timely or possible in the case of a decommissioned TPS, thus might compromise patient care, especially for patients who need an emergent treatment. If still existing, archived treatment plan data are large and typically contained within increasingly outdated technology, such as tapes, for historic or economic reasons. Retrieving archived treatment planning data is technically challenging due to outdated hardware/software/operating systems, incompatibility of DICOM data, inability to read DICOM data, or significant time required to locate the tape with the correct patient information. For emergent treatments, a comprehensive plan report that is easily accessible is critical for patient care.

Given the aforementioned arguments, I am for the proposition, which is also a consensus recommendation from members in Medical Physics Practice Guideline (MPPG) 11, charged with creating minimum practice guidelines for plan and chart reviews in external beam radiotherapy and brachytherapy. A standardized and comprehensive plan report can facilitate plan quality assurance prior to treatment (e.g., initial plan check from therapists) and during treatment (e.g., weekly chart check) from multiple team members in the radiation oncology department. A PDF file format for the plan report can serve as a durable record to mitigate the hazards of relying solely on proprietary treatment planning data. With the advancement in automation, creating a treatment plan report will be and is becoming less laborious.

As a final disclaimer, I am chairing the above‐mentioned MPPG 11 committee, which is formed by a group of members from academic centers and community practice centers with a wide spectrum of clinical experience using both integrated RO‐EMR with TPS and nonintegrated RO‐EMR with TPS.

### 2.B Dr. Arthur Olch

Any quality assurance (QA) procedure the physicist implements should be chosen because it efficiently and effectively mitigates errors. These procedures should address specific important failure modes and use cases. The proposition suggests that creation of a treatment plan report is a critical QA step, rising to the level of a minimum practice standard. Presumably, the report would be used for pretreatment and possibly during‐treatment data consistency checks. Secondarily, it could be used by people either inside or outside the radiation oncology department without access to the TPS to be able to review the plan. It could also be used to inform other radiation oncology departments if the patient were to be retreated elsewhere. Finally, such reports could serve as the long‐term archive of the plan information in the event the TPS were decommissioned and the digital data lost. I will discuss the effectiveness of the proposal for each of these use cases, but first, let us consider the time element.

It is important to avoid creating QA procedures that are ineffectual yet take time away from more effective procedures. The proposition suggests that for every patient, a treatment plan report be created so that it is available for personnel to view. There are various ways to create such a report, some are more automated than others. Creating and storing a PDF from a treatment plan generally can take at least 10 min per patient, much longer depending on the manual effort needed for screen captures and document assembly. Performing this process for hundreds of patients per year can take about 50 h of physicist/dosimetrist time at a minimum.

There are two scenarios where such a report could be used for QA, depending on whether there is an integration of the RO‐EMR/TPS with the treatment delivery systems (TDS). In the scenario where they are independent, data in the TPS must eventually be transferred to the database that talks to the TDS. There is an opportunity for data to be incorrectly transferred, and thus, the data that gets to the TDS must be checked for accuracy. This is done as a pretreatment check and for some centers or some special procedures prior to every treatment fraction. Consider that there are thousands of linac parameters, including gantry, couch, collimator angles, jaw and MLC positions, and MUs per segment for all beams in the plan. No one can check all of those effectively by manual comparison of values in a PDF. Automated methods are a better alternative which are available but not yet widely used. Admittedly, having disjointed systems introduces hazards which an integrated system largely avoids. In the integrated system, the TPS and TDS share a common database with only the most updated synchronized version of the data. A data integrity check is performed at the treatment delivery console each time plan data are sent to its local computer, a much more reliable way to check that transfer than a manual one. Some may use the plan report to confirm the plan has not been inadvertently changed since physician approval. A more effective method is to implement departmental procedures that avoid this. A treatment plan report should not be mandated when other more effective QA methods are available.

It is probably fair to assume that outside institution requests do not come often for most radiation oncology clinics. Also, very few requests are made at my institution by someone outside my department to view plan data. For those rare cases, one could screen‐capture dose distribution data from three dimensional views, which are interpretable by a nonradiation oncologist who made the request. In the case where the patient needs to be retreated at another facility, sending a PDF is an unsatisfactory way to communicate the 3D dose that was already delivered. Instead, DICOM data that contain CT images, plans, contours, and dose distributions should be transferred to the requesting party, so the dose can be comprehensively taken into account. I have not had a single situation in many years of transferring and receiving such data where it could not be done, and if it were a problem, a PDF could be sent. There is no need to create this report for 100% of patients. For the situation where the TPS gets decommissioned, all DICOM data should first be exported to the new system which ensures continuity. The time and cost of this export would be incurred once every great while (if at all) and should be considered part of the cost for new TPS system implementation and commissioning. Some TPS vendors even offer this data restore and transfer as part of the on‐board package.

Based on these considerations, creating a PDF for every patient is both inefficient and ineffective. At best, creation of a plan PDF for unusual situations might be warranted. There are other procedures which can better solve the problems that have been identified. Certainly the need is minimal for integrated systems. Furthermore, minimum practice standards are designed to be co‐opted by regulators who then will enforce them. Being required to create treatment plan reports for every patient or violate governmental regulations would be an onerous situation.

## 3 REBUTTAL

### 3.A Dr. Ping Xia

I agree in principle with the opening statement from Dr. Olch: “Any QA procedure the physicist implements should be chosen because it efficiently and effectively mitigates errors.” However, many of our practice standards are dictated by accrediting bodies or regulations. There are good reasons for these mandates, which have been carefully considered from experts in the field. Spending 5–10 min to create a treatment plan report is an efficient QA procedure when compared to many other QA procedures we perform.

Most of treatment parameters from a plan are electronically transferred from the TPS to the RO‐EMR system which then communicates to the treatment delivery console. Checking that the data transfers correctly is only a small aspect of plan review, and checking plan integrity, which is defined in MPPG11, is a more important part of plan review. Creating a plan report for QA does not mean that thousands of parameters should be checked manually. Experienced radiation oncology personnel know which key parameters to check for potential errors that could be inadvertently made in planning. These key parameters, such as simple indicators of beam geometry monitor units, locations of the iso‐center, do not constitute thousands or even hundreds on the most complex plans. Some errors cannot be caught exclusively by the integration of the RO‐EMR and the planning system. For example, a wrong iso‐center association with treatment fields/imaging, a wrong energy used for the plan, or a wrong site planned for the patient, just to name a few. Given many potential scenarios, multiple layers of efficient initial plan checks/reviews are important and necessary with different team members (e.g., physicists, physicians, and therapists). Subsequently, a periodic plan check (e.g., weekly) during treatment is required by regulations and accreditation standards. Using a plan report significantly improves the efficiency of these plan and chart review processes, especially for team members who do not use a TPS routinely. Depending on the complexity of a plan and how comprehensive of a plan report, a physicist can conduct an initial plan check using the plan report efficiently and effectively. If a question arises, or the plan is particularly complex, the physicist can directly access the TPS for further investigation.

In theory, I also agree with Dr. Olch that automation is better than a manual check. In reality, automated programs are mostly rule based and require continuous adaptation to ever changing clinical practices. Published studies,^3^, ^4^, ^5^ including one from my institution,^5^ showed that hybrid automated checks and manual checks catch errors and are important for patient safety.

Lastly, integrated RO‐EMR and treatment planning systems have advantages and disadvantages. With the integration, the plan file does not need to be imported into another system and the concern about file transfer integrity is eliminated. However, without the separation between a planning and delivery system, there is a risk that a plan could be un‐intentionally changed. As pointed out by Dr. Olch, a process must be established to check each time plan data are sent to the treatment delivery console. Without integration, the treatment file is checked after being imported and locked in TDS to prevent incidental changes during the course of treatment. In conclusion, creating a plan report is an effective and efficient QA process. The combination of an automated and manual review of a treatment plan and patient chart will remain one of the many QA processes we routinely perform.

### 3.B Dr. Arthur Olch

Dr. Xia correctly points out the importance of plan QA by referencing the findings in the RO‐ILS report. The question we are raising here is what is the most effective and efficient method to perform it. The first situation mentioned is that radiation oncology staff members who need to perform plan QA may not understand how the TPS software interface works. After all, everyone can view a PDF. However, those charged with QA’ing a treatment plan would be dosimetrists or physicists, they surely know how to navigate the TPS. A comprehensive evaluation of the treatment plan would never be possible with a PDF and even providing a PDF might be a bad idea since it could be a disincentive to actually using the TPS to review the plan. Therapists might need to view and confirm plan parameters either pretreatment or during weekly checks but that does not require access to the TPS, these parameters can be found in the RO‐EMR which everyone should have access to and have been trained to use. The next scenario that was mentioned in support of a PDF plan report is the case where the TPS is inaccessible for some reason. TPS downtime for upgrades is planned and if PDFs were needed just at that time, they could be created in advance just for that occasion. The third scenario cautioned that just‐in‐time creation of PDFs or DICOM data might not be timely for outside transfer especially for emergent patients. Would you want to create PDFs for 100% of your patients just so you would not have to prioritize a DICOM data transfer to an outside center which generally takes <15 min, and which is needed only infrequently? Next, an argument is made that in the event archived TPS data were needed for an emergent treatment, it could be challenging to get it in a timely fashion, making the plan report a requirement. Again, how often is archived data needed for an emergent treatment where you did not have a day to retrieve the data? Why are data being archived in a way that makes it so hard to retrieve in a timely manner? In this era where terabytes of data storage are common, it seems prudent to either keep all patient data on‐line or move away from archival systems that cannot provide timely restoration. This scenario does not lead to a compelling reason to mandate the creation of plan report PDFs for 100% of your patients.

In summary, relying on a plan report PDF that takes considerable time to create for 100% of patients does not address any problems that cannot be solved with other methods or processes. When used for QA instead of direct TPS review, a false sense of safety may result. PDFs may have a limited QA role to play for nonintegrated systems, but for integrated system, they serve no purpose. In a bygone era where data storage or export were cumbersome or where the RO‐EMR or TPS were not networked, PDFs might have been useful. In 2020, radiotherapy centers should have the tools they need to use digital systems and well thought out processes to address all the concerns mentioned by Dr. Xia and should not be mandated to continue to use PDFs.

## CONFLICT OF INTEREST

The authors declare no conflict of interest.
